# First Report of Concomitant Tinea Faciei and Pityriasis Folliculorum: A Dermatomicrobiological Rarity

**DOI:** 10.7759/cureus.3017

**Published:** 2018-07-20

**Authors:** Hari Pankaj Vanam, Kalyani Mohanram, Siva Rami Reddy K, Sri Shilpa Poojari, Anuradha P.R, Venkataramana Kandi

**Affiliations:** 1 Department of Mycology Division-Microbiology, Bhaskar Medical College and General Hospital, Hyderabad, IND; 2 Department of Microbiology, Saveetha Medical College, Chennai, IND; 3 Department of Dermatology, Venerology and Leprosy, Bhaskara Medical College, Hyderabad, IND; 4 Department of Dermatology, Venerology and Leprosy, Dr Patnam Mahender Reddy Institute of Medical Sciences, Hyderabad, IND; 5 Department of Microbiology, Bhaskara Medical College and General Hospital, Hyderabad, IND; 6 Department of Microbiology, Prathima Institute of Medical Sciences, Karimnagar, IND

**Keywords:** pityriais folliculorum, tinea faciei, demodicosis, dermatophytosis, trichophyton mentagrophytes complex, trichophyton mentagrophytes var interdigitale, ectoparasitic infestation, t. interdigitale

## Abstract

Tinea faciei (TF) is a common dermatomicrobiological condition caused by dermatophytes involving the skin of the face but not the mustache and beard (Tinea barbae). It poses a diagnostic dilemma with its atypical clinical presentation. Pityriasis folliculorum (PF) is a dermatological condition that results in rosacea-like skin eruptions. It was previously associated with a human ectoparasitic infestation. *Demodex* mites (*Demodex folliculorum*) is a group of obligate parasites that live on the skin of mammals. These mites have been associated with various dermatological disorders, clinically termed as demodicosis. Insects have been described as potential vectors that can carry various microorganisms and especially spores of fungi. Hence, infestation by such insects may aggravate the already present skin condition, leading to secondary infections. There has been a change in the trend of dermatophytosis worldwide and infections caused by *Trichophyton mentagrophytes var. interdigitale *(*T*. *interdigitale*) are increasing. Hence, there is an urgent need for a thorough investigation of an infectious etiology among various skin disorders. This is the first report of concomitant Tinea faciei and Pityriasis folliculorum involving facial skin.

## Introduction

Tinea faciei (TF) is a skin disease that poses a diagnostic dilemma among dermatologists because of its atypical presentation. All the three genera of dermatophytes, including *Trichophyton*,* Epidermophyton*, and *Microsporum,* have been associated with TF [1]. The *Trichophyton mentagrophytes* (*T*. *mentagrophytes*) complex has been the most frequent cause of TF followed by *Microsporum gypseum*and *Microsporum canis* (*M*.* canis*). Among children, *T*.* tonsurans, T*. *violaceum,* and *M*. *canis* were frequently reported in TF [2].

Pityriasis folliculorum (PF), on the other hand, is a clinical condition that is characterized by the presence of rosacea-like eruptions and a high density of *Demodex *mites either in one follicle of hair or with extra-follicular involvement [3]. Demodicosis is the term given to all cutaneous diseases caused by an ectoparasite belonging to the genus *Demodex* mite. *Demodex* mites feed on the sebum and skin cells and, hence, they localize in and around sebaceous glands where there is sebum production. Typically, in 23%-100% of the human population, *Demodex *mites are localized on the face, including cheeks, nose, chin, forehead, temples, eyelashes and eyebrows, the balding scalp, neck, and ears. It has been described as a vector carrying various microorganisms and especially spores of dermatophyte *M*.* canis* [4-5].

There has been a change in the trend of dermatophytosis worldwide and *T*.* mentagrophytes* has evolved as a co-dominant contagion that was previously underestimated in human dermatological conditions. Speciating the isolate has taxonomical and epidemiological significance since human-adapted *T*.*mentagrophytesvar*. *interdigitale *(*T*. *interdigitale*) is morphologically indistinguishable from the zoophilic counterpart. It was noted that the zoophilic *Trichophyton* species of *Arthroderma benhamiae*, of which small rodents (guinea pigs) are reservoirs, overlap with the zoophilic *T*.* interdigitale *isolates [6]. Hence, any glabrous skin infection by *T*.* mentagrophytes *complex and cases with co-infections warrant a thorough investigation using both mycological and molecular tools. Antimicrobial susceptibility testing assumes increased significance when treating patients with significant co-morbidities.

We present the first report of microbiologically confirmed TF caused by *T*. *interdigitale* with a concomitant *Demodex* mite infestation causing PF.

## Case presentation

**Figure 1:**
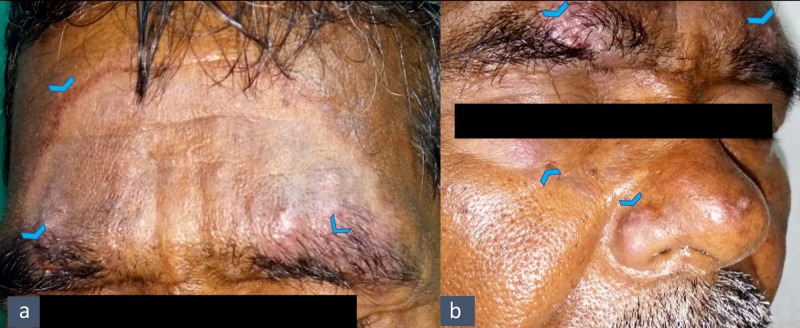
Description of lesions of Tinea faciei and Pityriasis folliculorum a. Annular erythematous lesions on the forehead with sharp margins and raised edges (arrowheads); b. Scaly plaques with papular to papulopustular lesions involving both the eyebrows, the nose and the left cheek (arrowheads)

A 60-year-old male patient presented to the dermatology outpatient department (OPD) with a two-month history of diffuse facial erythema, itching, and burning sensation. Raised skin lesions were noted on the forehead, nose, and left cheek. He also complained about the exacerbation of lesions upon exposure to the sun. On dermatological examination, well-defined annular erythematous lesions over the forehead, with a sharp margin and a raised edge, and scaly plaques with papular to papulopustular lesions involving both the eyebrows, the nose, and the left cheek were revealed, as shown in Figure 1.

The patient gave a history of myocardial infarction and cardiac surgery and was on antidiabetic and antihypertensive drugs. He gave a history of sharing linen and admitted to the habit of sleeping outside the house in the open air during the summer season. The patient also informed about a rodent infestation in his immediate surroundings and frequent animal contact. A history of previous nail infection on the right great toe, which was otherwise normal upon examination, was noted. No other concomitant infections were observed. The patient denied any application of topical corticosteroids or any self-medication. An intramuscular injection of dexamethasone was given 10 days before by a local practitioner, which gave temporary relief from erythema and tingling.

Processing of the specimen for a mycological/microbiological examination

Skin scrapings were taken under aseptic precautions from the extending and raised margins of the lesion on the forehead. Potassium hydroxide preparations revealed more than five *Demodex folliculorum* mites measuring ~ 0.3-04 mm in a scraping of 1 cm^2^ area along with a moderate number of hyaline septate hyphae, with a few hyphae breaking into chains of arthroconidia under 40X magnification of the microscope.

**Figure 2:**
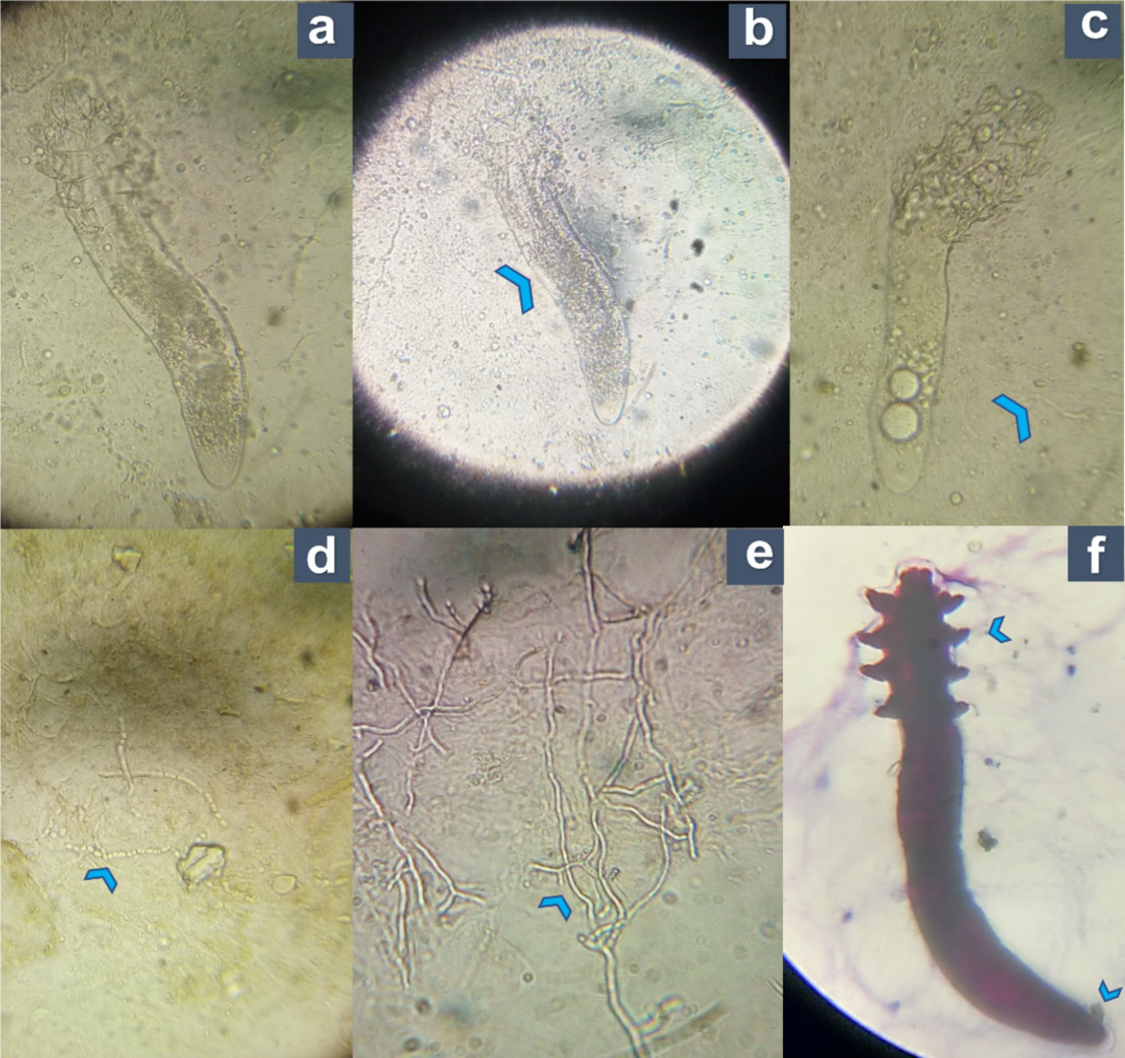
Direct microscopy using KOH and modified acid-fast stain showing hyphal elements and Demodex mites a. Direct 20% KOH preparation of the skin scrapings (extrafollicular involvement) showing *Demodex* mites (40X); b. *Demodex *mites in KOH mount showing the posterior segment with secondary striations (arrowhead); c. *Demodex* mite seen along with fungal elements in skin scrapings (arrowhead); d-e. KOH preparation of the skin scrapings showing a moderate number of hyaline septate hyphae, few breaking into chains of arthroconidia (arrowheads); f. Modified acid-fast staining showing *Demodex folliculorum *mite stained in blue color (40X). The mite is seen with two fused segments. The first segment has four-pairs of legs (arrowhead) and the posterior segment is longer, with a round opisthosomal end (arrowhead). KOH: potassium hydroxide

The mite was semitransparent, with an elongated body formed by two fused segments. The first segment had four pairs of legs and a hairless and colorless body surface. The second or posterior segment had secondary striations. The longer posterior segment at the opisthosomal end was round, differentiating it from *D. brevis, *which has a short and pointed opisthosomal end, as shown in Figure 2 [5].

Repeated slit skin smear examinations were negative for acid-fast bacilli. A diagnosis of Pityriasis folliculorum with concomitant dermatophytosis (Tinea faciei) was made.

The specimen was later inoculated on a dermatophyte test medium (DTM) agar base with a dermato supplement and Sabouraud’s dextrose agar (SDA) with 50 mg chloramphenicol and 0.05% cycloheximide (HiMedia, India) and incubated in ambient air at 28°C (Biological Oxygen Demand/Biochemical Oxygen Demand (BOD) incubator).

Growth started to appear after five days of incubation, which was later subcultured on to potato dextrose agar (PDA).

Routine slide culture, microplate culture, and adhesive tape preparations for the phenotypic identification of the dermatophyte fungus along with a urease test and a hair perforation test were performed.

Phenotypic identiﬁcation and morphological description of T. mentagrophytes

The surface texture of the colonies on DTM agar base initially showed downy, later turning into pleomorphic and powdery, growth with a flat surface after two weeks of further incubation. When subcultured on to PDA, a pale brown to buff or yellow surface producing a characteristic stellate powdery surface growth was observed after two weeks of incubation. The reverse showed a dark red pigment rather than the usual light yellow or brown-tan color. When grown on cornmeal agar (CMA), there was no reverse pigment.

Macroconidia were demonstrated only in the early colonies grown on the DTM agar base and PDA, which were noted to be thin, smooth-walled and cigar-shaped, with a few of them bearing narrow attachments to the vegetative hyphae. Microconidia were spherical to pyriform and arranged in grape-like clusters.

**Figure 3:**
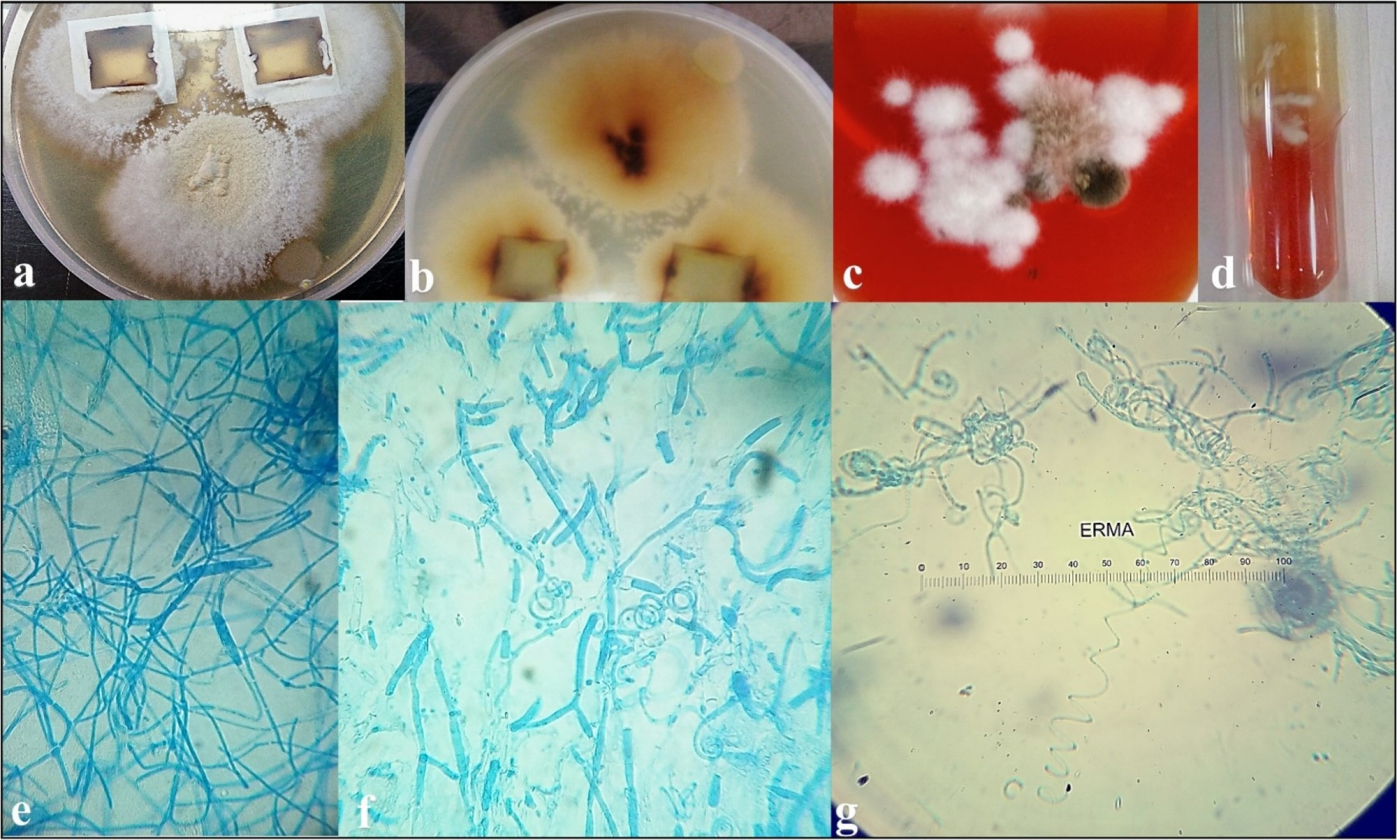
Cultural and phenotypic attributes of T. mentagrophytes complex a. Microplate culture on PDA after two weeks showing stellate, light brown, powdery growth; b. Reddish reverse pigmentation; c. Growth on DTM agar base initially showing downy colonies; d. Urease positive; e. Lactophenol cotton blue mount of fresh colonies of *T. mentagrophytes* complex showing macroconidia; f. Peridial hyphae and scattered microconidia; g. Abundance of spiral hyphae and a few microconidia as observed from mature colonies.

Older cultures exhibited pleomorphism with antler-like hyphae and peridial hyphae with the terminal branches bearing two or three cells with an abundance of spiral hyphae, as shown in Figure 3.

The isolate was positive for the urease and hair perforation tests and was phenotypically confirmed as *T. mentagrophytes. *Due to its variable morphological and cultural attributes, the isolate was submitted for confirmation and antifungal susceptibility data to the national reference centre, National Culture Collection of Pathogenic Fungi (NCCPF), an Indian Council of Medical Research (ICMR), New Delhi, sponsored center at the Postgraduate Institute of Medical Education and Research (PGIMER), Chandigarh, India.

Confirmation of Trichophyton interdigitale by molecular identification

Molecular identiﬁcation of the culture with submission identification (ID) nccpf IL-2878 was performed at the national reference center by amplifying the DNA and sequencing the internal transcribed spacer (ITS) region of rDNA using the ITS1 and ITS2 primers. *T*.*interdigitale* was conﬁrmed by comparing the sequence in Centraalbureau voor Schimmelcultures (CBS), Utrecht, The Netherlands, the Dermatophytes database [7], and the National Center for Biotechnology Information (NCBI) GenBank database, respectively, which showed a 99.61% similarity with the type strain CBS 130940 *T*.*interdigitale.*

GenBank direct submission and accession ID: The T.*interdigitale* ITS sequence was deposited in GenBank ITS databases and published in the NCBI database on 16/5/2018 with accession number MH327513 and name *T*.*interdigitale* - IL2878/333.

Antifungal susceptibility testing 

Antifungal susceptibility testing (AFST) was performed following the Clinical Laboratory Standards Institute (CLSI) guidelines (M38-A2). The broth microdilution (BMD) method was used to determine the minimum inhibitory concentrations (MICs) of the test isolate against various antifungal drugs, which included itraconazole, sertaconazole, terbinafine, griseofulvin, and amorolfine. All the antifungal drug powders were reagent grade and were procured directly from Sigma Aldrich (Missouri, United States) by PGIMER/NCCPF, Chandigarh, India. RPMI-1640 (HiMedia, India) with L-glutamine, without bicarbonate, buffered at pH 7.0 with 0.165 M morpholine propane sulfonic acid (MOPS buffer) was used for inoculum preparation and to perform broth microdilutions. All the antifungal powders tested were dissolved in dimethyl sulphoxide (DMSO). Three quality control strains, including *Candida parapsilosis* American Type Culture Collection (ATCC) 22019, *Candida krusei *ATCC 6258, and *Aspergillus flavus* ATCC 204304 were included. Inoculum suspension and dilutions were performed as described by Adimi et al. [8]. Endpoint determination and interpretation of MICs was done according to CLSI M38-A2 and from the recent AFST studies [9-10].

The results of the antifungal susceptibility testing of five antifungal agents are shown in Table 1.

**Table 1:** Interpretation of antifungal susceptibility testing using the broth microdilution technique

Antifungal agent tested	Dilution range in μg/mL	MIC in μg/mL	Interpretation
Amorolfine	0.0078 - 4 μg/mL	0.0156	Most effective
Itraconazole	0.016 - 6 μg /mL	0.0625	Most effective
Terbinafine	0.004 - 4 μg /mL	0.0078	Most effective
Sertaconazole	0.016 - 16 μg /mL	0.125	Effective
Griseofulvin	0.25 - 128 μg /mL	8	Least effective

Antifungal and acaricide therapy

The patient was treated with oral terbinafine 500 mg once daily for four weeks. Combination topical therapy involving a cleanser containing selenium sulfide 2.5% lotion followed by the application of 2% sertaconazole twice daily and 1% metronidazole gel for three weeks was initiated. The patient had an uneventful recovery.

## Discussion

TF is one among several dermatological conditions presented by the patients visiting the dermatology clinics. Due to the complexity of the facial anatomy and because of the atypical presentation of the lesions that mimic many other dermatological disorders (atopic dermatitis, bacterial infections, cutaneous candidiasis, granuloma annulare, lupus erythematosus, and Demodex folliculitis), TF poses a diagnostic difficulty for dermatologists. Up to 70% of TF cases are misdiagnosed, resulting in delayed diagnosis and initiation of treatment. The application of a steroid ointment could be detrimental, which further modifies the TF lesions, resulting in diffuse erythematous patches, plaques, scaling, papular or pustular lesions with hyperpigmentation, and darkening of skin known as Tinea incognito. Of the TF cases, 85% were also observed to involve the nails and, hence, a thorough clinical evaluation is important to minimize the spread [11]. Tropical and humid conditions favor both TF and PF; sun exposure can exacerbate the symptoms and both conditions showed an evidence of person-to-person spread and recurrences [11,3-5]. Therefore, in diagnosing these two clinically atypical forms of dermatosis, one should have a high index of suspicion while considering the potential role of follicular mites in cutaneous diseases and coinfections with dermatophyte fungi [4].

The early descriptions of follicular mites were given by Henle, Berger, and Simon in the 1880s. Akbulatova, in 1963, identified two-subspecies and named them *D*. *folliculorum longus* and *D*.* folliculorum brevis*. *D*.* folliculorum longus *was commonly associated with lesions on the face and *D*.* folliculorum brevis *has been noted to involve the neck, chest, and other areas of the body [12].

Patients with more than five mites per low-power field, in one follicle, in one skin scraping, or in one-cm^2^ area by standardized skin surface biopsy (SSSB), is considered as diagnostic for significant infestation [13]. The first reports of demodicosis caused by *D*. *folliculorum *associated with allergic manifestations were reported from the city of Kolkata in India during 1975-76 [12]. A case of unilateral demodicosis of the face, mimicking Hansen's disease was reported from Pune, India [14].

Reports of infestations of *Demodex* mites and their association with various skin disorders, including follicular dyskeratosis with light pigmentation and rosacea-like dermatosis, dates back to a century [12,15].

It was also hypothesized that *Demodex *mites may act as potential vectors in the transmission of pathogenic microorganisms like the Lepra bacilli (*Mycobacterium leprae*), tumor-inducing viruses, *M*. *canis*, and *Staphylococcus aureus *[15].

Ayres and Ayres, in 1961, coined the term “Pityriasis folliculorum” for the lesions presenting as acne, pustules, superficial vesicles, rosacea-like skin eruptions, and the presence of *D*.* folliculorum* mites [16]. It was also observed that the lesions showed dramatic resolution after applying the ‘Danish ointment’ (a sulfur and beta-naphthol containing vaseline preparation) [16].

From the observations of the current study where mites were found surrounded by the fungal hyphae, it could be possible that the *Demodex *mites may feed on the dermatophyte fungus on the facial skin and, hence, could alleviate the effect of TF. On the other hand, considering the role as a vector, *Demodex *mites themselves can be implicated in spreading pathogenic microorganisms and resulting in exacerbations and secondary infections.

Fungi belonging to the phyla* Ascomycota *and *Basidiomycota *are characterized based on the presence of sexual and asexual structures in their life cycles. They are called as teleomorphs when sexual reproductive structures are present, anamorphs when asexual reproductive structures are seen, and holomorphs when both the sexual and asexual structures are seen. Based on where they inhabit, dermatophytes can be characterized as geophilic (present in the soil), zoophilic (animals), and anthropophilic (cause human infections).

The *T.* *mentagrophytes *species complex comprises the most pleomorphic dermatophytes and includes *T*. *mentagrophytes* (teleomorph - *Arthroderma vanbreuseghemii*), *T*.* interdigitale *(no teleomorph reported till date), *T*.* simii* (teleomorph - *Arthroderma simii*), *T*.* erinacei* (no teleomorph reported till date), and *Arthroderma benhamiae*. *T*. *interdigitale *is unique in being anthropophilic, and the others are either zoophilic or geophilic.

The human-adapted anthropophilic *T*.* mentagrophytes* is named as *T*.* mentagrophytes* *var*. *interdigitale,* and it differs both in its clinical presentation and in its morphological features from their zoophilic counterparts. *T*. *interdigitale *has become a growing concern in India and elsewhere, causing inflammatory tinea, replacing the more common contagion *T*.* rubrum*. The anamorphic classification of species *T*. *mentagrophytes* is still confusing and is a matter of debate. Since the use of anamorphic and teleomorphic nomenclature is confusing, species showing polymorphism are, therefore, summarized as the *T*.* mentagrophytes* species complex [17]. Experimental studies have confirmed that *T*. *interdigitale *should be considered as a human-adapted species, which could have evolved from the teleomorph *Arthroderma vanbreuseghemii* [18].

Due to the evolution of *T*. *interdigitale* from the zoophilic dermatophyte *Arthroderma vanbreuseghemii*,* *reports of infections with *T*.* interdigitale* and its association with TF in humans is rarely reported [19].

The dermatological presentations during concomitant skin disorders may be atypical as in the present scenario attributed to the intense cell-mediated immune response against the follicular mites and fungal elements. Laboratory diagnostic tests should be appropriately applied to confirm the causative agent and initiate appropriate therapeutic measures for better patient care and management.

The AFST data of the *T. interdigitale* isolate from the present report showed good in-vitro activity against terbinafine, amorolfine, and triazoles but keeping in view the patients' co-morbidities and possible drug interactions, triazoles like itraconazole were not recommended for systemic therapy. Sertaconazole was chosen for its potent topical antifungal activity and its preferential use in cutaneous mycoses by its unique binding affinity to non-sterol lipids in the fungal cell wall along with inhibiting the ergosterol synthesis pathway. Additional attributes of sertaconazole include the dermal retention and attainment of fungicidal concentration in the stratum corneum thus potentiating an increased permeability when given in higher concentrations [20].

Although few studies have implicated demodicosis and TF as frequent causes of dermatological disorders from India and around the world, this appears to be the first instance where demodicosis was demonstrated in an extrafollicular area with concomitant dermatophytosis.

## Conclusions

A laboratory diagnosis of dermatological lesions assumes greater significance in the event of concomitant skin disorders. The extra-follicular infestation of *Demodex* mites and the occurrence of TF caused by *T*. *interdigitale *signifies the changing trends in the etiology and clinical presentation of dermatological disorders. This is a true case of the convergence of two different clinical entities. Clinical suspicion combined with laboratory isolation and the identification of the etiological agents along with the results of antimicrobial susceptibility testing could be crucial for the dermatologists/clinicians to make appropriate treatment recommendations for a favorable clinical/patient outcome. Molecular and epidemiological studies are recommended for characterizing the *T. mentagrophytes *speciescomplex.
